# The role of informal dimensions of safety in high-volume organisational routines: an ethnographic study of test results handling in UK general practice

**DOI:** 10.1186/s13012-017-0586-8

**Published:** 2017-04-27

**Authors:** Suzanne Grant, Katherine Checkland, Paul Bowie, Bruce Guthrie

**Affiliations:** 1 Population Health Sciences, School of Medicine, University of Dundee, The Mackenzie Building, Kirsty Semple Way, Dundee, DD2 4BF UK; 2 Health Policy, Politics and Organisation (HiPPO) Research Group, Centre for Primary Care, Institute of Population Health, University of Manchester, 5th Floor, Williamson Building, Oxford Road, Manchester, M13 9PL UK; 3 NHS Education for Scotland, 2 Central Quay, Glasgow, G3 8BW UK

**Keywords:** Test results handling, General practice, Primary care, Organisational routines, Safety, Ethnography, Qualitative

## Abstract

**Background:**

The handling of laboratory, imaging and other test results in UK general practice is a high-volume organisational routine that is both complex and high risk. Previous research in this area has focused on errors and harm, but a complementary approach is to better understand how safety is achieved in everyday practice. This paper ethnographically examines the role of informal dimensions of test results handling routines in the achievement of safety in UK general practice and how these findings can best be developed for wider application by policymakers and practitioners.

**Methods:**

Non-participant observation was conducted of high-volume organisational routines across eight UK general practices with diverse organisational characteristics. Sixty-two semi-structured interviews were also conducted with the key practice staff alongside the analysis of relevant documents.

**Results:**

While formal results handling routines were described similarly across the eight study practices, the everyday structure of how the routine should be enacted in practice was informally understood. Results handling safety took a range of local forms depending on how different aspects of safety were prioritised, with practices varying in terms of how they balanced thoroughness (i.e. ensuring the high-quality management of results by the most appropriate clinician) and efficiency (i.e. timely management of results) depending on a range of factors (e.g. practice history, team composition). Each approach adopted created its own potential risks, with demands for thoroughness reducing productivity and demands for efficiency reducing handling quality. Irrespective of the practice-level approach adopted, staff also regularly varied what they did for individual patients depending on the specific context (e.g. type of result, patient circumstances).

**Conclusions:**

General practices variably prioritised a legitimate range of results handling safety processes and outcomes, each with differing strengths and trade-offs. Future safety improvement interventions should focus on how to maximise practice-level knowledge and understanding of the range of context-specific approaches available and the safeties and risks inherent in each within the context of wider complex system conditions and interactions. This in turn has the potential to inform new kinds of proactive, contextually appropriate approaches to intervention development and implementation focusing on the enhanced deliberation of the safety of existing high-volume routines.

## Background

The handling of test results (or ‘test results handling’) in general practice in the UK National Health Service (NHS) (e.g. for blood and urine tests, X-rays, and ultrasound scans) is a high-volume organisational routine that is both complex and high risk [1–4]. Test results are usually initially handled by a practice receptionist, but all must be checked by a clinician, usually either a general practitioner (GP) or a practice nurse. Many will then require subsequent action, such as a repeat test, a change in medication, patient review, referral or further investigation. Although information technology (IT) can automate parts of the system such as the delivery of results to the responsible clinician, multiple administrative staff members still contribute to ensuring that the appropriate clinician reviews the result and that any necessary action is completed.

To date, research examining the safety of test results handling has mainly focused on the quantification, assessment and management of errors and harm [5–7]. For example, a recent study found 17 potentially error-prone steps in the primary care laboratory results handling process [8]. Other studies have shown that errors can directly impact on care through duplication of services, unnecessary or delayed interventions, morbidity and mortality caused by treatment delay, and patient dissatisfaction and litigation [7, 9–12]. This mirrors wider trends in patient safety research and improvement initiatives known as ‘Safety-I’ [13], where the dominant focus is ‘measuring and managing’ [14] more negative dimensions of safety through evidence-based procedures and guidelines. While this research has improved understandings of how and why patients are harmed and has usefully informed evidence-based improvement practices, it has not consistently achieved expected improvements in safety [15]. This is likely because it is not always well suited to complex adaptive systems such as healthcare, where interactions between people and technology are not always linked in a predictable manner [16–21]. Furthermore, while adverse events are not uncommon, things usually go right for the vast majority of health care delivery [22].

Complementary ways of understanding and improving safety in complex healthcare settings have therefore emerged such as ‘exnovation’ [22], ‘positive deviance’ [15, 23–25] and ‘Safety-II’ [13]. These new approaches aim to better understand the underlying dimensions of *safe* practice by examining the key features of everyday care provision (e.g. inter-professional collaborative work), and the adjustments and trade-offs made my professionals when faced with less predictable risks [26, 27]. While these prioritisation decisions tend to be informal, implicit and habitual, healthcare improvers have become increasingly interested in surfacing this underlying expertise as an important quality and safety improvement resource. Such approaches have demonstrated that informal, in situ solutions to safety are more likely to be readily acceptable and feasible to implement more widely as complex interventions because they are sensitive to the complexities of everyday healthcare delivery [28].

One context where this approach has not yet been widely applied is high-volume organisational routines such as test results handling. An organisational routine can be defined as ‘a repetitive, recognizable pattern of interdependent actions involving multiple actors’ [29]. While organisational routines can act as normative structuring devices that co-ordinate what individuals *should* do (‘formal routines’), studies have also highlighted the important role of less visible elements of routines as they are practiced every day (‘informal routines’) as the key components of patient safety [30–34]. Focussing on informal high-volume organisational routines therefore has the potential to identify the range of prioritisation decisions that healthcare teams make to achieve safety and mitigate risk across different parts of a routine, which can usefully inform the design and development of complex interventions to improve the safety of test results handling more widely [35].

This paper ethnographically examines the informal dimensions of test result handling routines in the achievement of safety in UK general practice and explores how these findings can best be developed for wider application by policymakers and practitioners.

## Methods

### Research setting

The study was conducted in the NHS in Scotland and England from January 2011–April 2014 using a multi-site ethnographic design across 8 urban and rural general practices. Practices were purposively selected on the basis of their size (smaller [<~7000 patients] or larger), location (urban or rural) and socioeconomic deprivation of the population served (affluent, mixed or deprived) (Table 1).

**Table 1:** Study practice characteristics

Practice number	Country	Practice size	Practice location	Practice socioeconomic deprivation
1	Scotland	~4000	Urban	Mixed
2	Scotland	~9000	Urban	Deprived
3	Scotland	~5000	Urban	Mixed
4	Scotland	~8000	Rural	Affluent
5	England	~5000	Urban	Mixed
6	England	~6000	Rural	Mixed
7	Scotland	~9000	Urban	Deprived
8	Scotland	~8000	Rural	Affluent

### Data collection procedures

Data collection was in two phases, with an in-depth ethnographic study conducted in practices 1–4 over a 24-month period in 2011/12. This was followed by a more focussed ethnographic study in practices 5–8 in 2013/14 involving 1 week of intensive fieldwork per practice focussing on high-volume safety critical processes identified in the first four practices. Fieldwork across all eight practices focussed on specific organisational routines, including repeat prescribing, data coding and document handling (including test results and hospital letters). Data collection combined non-participant observation of the everyday practices of team members with interviews and documentary analysis. The aim was to develop a rich and detailed ‘thick description’ [36] of each case study practice and the similarities and differences across all eight cases. SG undertook 1787 h of ethnographic fieldwork from January 2011 to April 2014. Informed consent was obtained from each practice team member prior to fieldwork commencing, and it was explained to informants that the researcher was interested in learning about the organisational culture, systems and processes of the practice and not in assessing individuals’ performance. Fieldwork was undertaken with clinical, managerial and administrative staff during normal working hours in reception areas, administrative back offices, consulting rooms, meeting rooms, coffee rooms and corridors. Detailed handwritten fieldnotes were made during observation and later written up more fully and transcribed for coding. Narratives were elicited from staff by the researcher while they worked, asking them to talk through what they were doing as they conducted their everyday work. Documentary analysis of relevant written protocols and patient information leaflets from each practice was also conducted.

Towards the end of fieldwork in each practice, a total of 62 semi-structured interviews were conducted with GPs, practice nurses, practice managers and receptionists. Interviewees were selected based on their involvement in high-volume organisational routines and each gave informed consent to participate. Interview topics included the interviewee’s role within the practice, practice organisational structure and culture, the key ways in which the workload was divided across the practice and why, interviewees' descriptions of each organisational routine that they were involved in, and how they collaborated with other practice team members. The interviews lasted 60 min on average and were recorded and transcribed verbatim.

### Data analysis

Analysis explored the issue of patient safety across diverse organisational routines, with the focus in this paper being test results handling. In particular, analysis drew on recent research focussing on the positive dimensions of safety [13, 22] and the informal work that was required to achieve safety and mitigate risk across different routines and contexts [32, 37]. A practice-based approach [38] to results handling routines was adopted that focussed on the differences and similarities between formal descriptions of results handling routines, and how these routines were informally enacted in everyday practice across different organisational settings [39, 40]. Fieldnotes and interviews were annotated with observational and theoretical notes as fieldwork progressed and were shared between the research team. The researcher (SG) read the interview transcripts to become familiar with the data. Preliminary themes were identified through scrutiny of initial transcripts and a coding framework was subsequently developed that was embedded in the data collected [41], with formal and informal safety practices used as sensitising concepts during the course of the analysis. The framework was applied and refined according to emerging themes across the eight practices as the fieldwork developed using NVivo 8 software. This constant comparative method continued until no further categories emerged.

## Results

The volume and complexity of results handling routines had increased over time in all eight practices in the study, and this was frequently described by team members as a safety issue in itself. In all practices, there was evidence of both formal organisational routines which participants were able to describe in the abstract and informal routines by which results handling was actually achieved in practice.

### Formal results handling routines

All eight practices had a formal organisational routine for the processing of laboratory, imaging and other test results that had been requested by a clinician. Practices received results on paper or electronically in different ways depending on how their local test provider worked, reflecting a general but incomplete move towards electronic systems in the UK during the period of the research. All practices used document handling software for managing results, which allows the passing of documents between different members of staff until all required actions were complete.

**Fig. 1:**
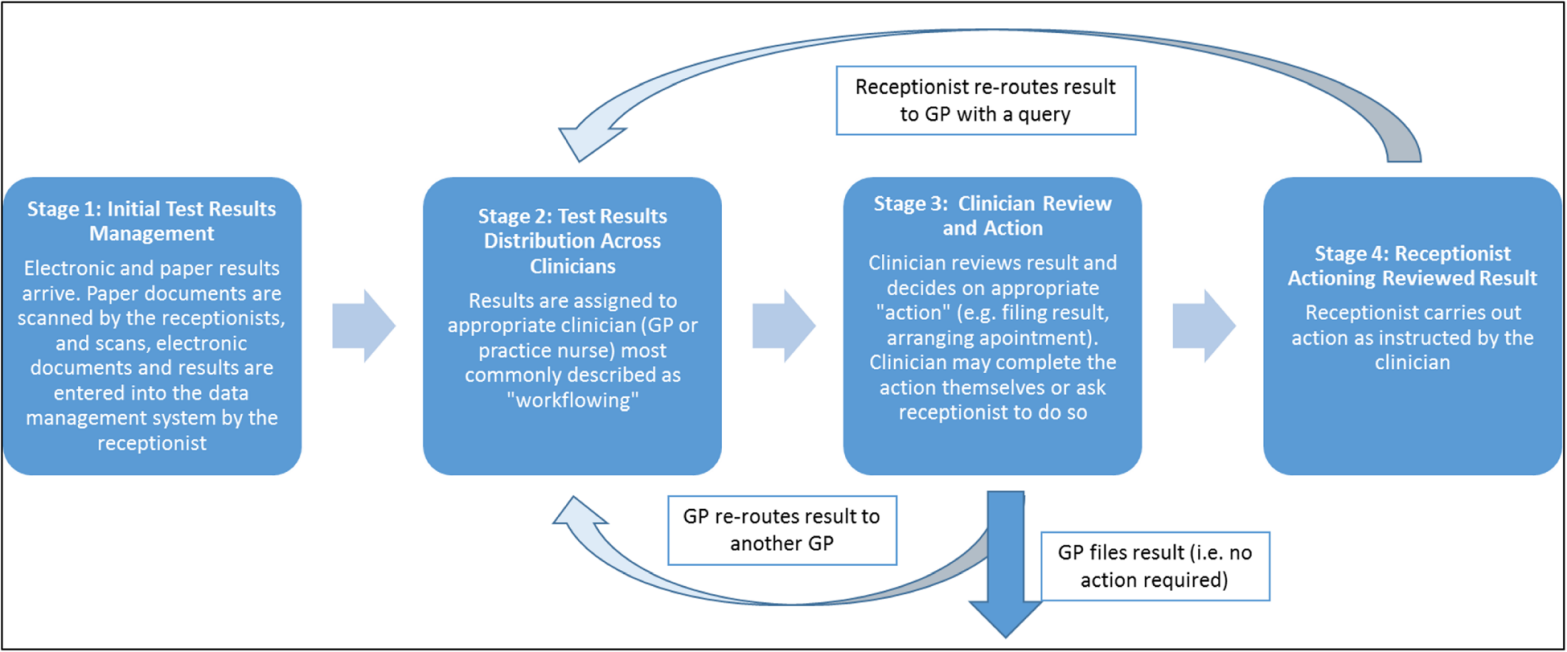
Key stages of the test results handling routine

Each practice operated similar formal systems for handling paper and electronic results. The four key stages in these formal routines are illustrated in Fig. 1. The process and language used to describe these stages were significantly influenced by the software design. Thus, for example, the terms ‘workflow’, ‘re-routing’ and ‘action’ were all embedded in the IT software and were also routinely used by staff to describe particular actions (e.g. ‘workflowing’ was used as a verb to describe the transfer of documents between team members). Five of the practices did not have a formal written protocol for their results handling routine. In the three that did (practices 4, 6 and 8), protocols focused on brief technical instructions such as operating the electronic document management system. In practice, however, the written protocols were rarely accessed by team members. For example, in practice 4, receptionists explained that while a protocol was available in the filing cabinet and on the IT system, they were only provided to new members of staff with the introductory training pack, with more experienced team members relying on a combination of basic technical knowledge and widely shared experiential knowledge. In spite of this lack of documentation, descriptions of the formal or expected steps in results handling routines were strikingly similar both within and between practices.

### Informal test results handling routines

When talking about the work of managing particular results, it was clear that the formal process required the application of considerable informal knowledge to manage the complexity experienced. The following sub-sections examine the characteristics of this informal knowledge and how it was employed in practice across the four stages of the routine (summarised in Table 2).

**Table 2:** Practice-level organisation of the distribution of test results

	Practice 1	Practice 2	Practice 3	Practice 4	Practice 5	Practice 6	Practice 7	Practice 8
Protocol	No	No	No	Yes	No	Yes	No	Yes
Stage 1: How was the initial management of results organised?	Single receptionist	Different person each day	Single receptionist	Same person for a week at a time	Different person each day	Different person each day	Same person for a week at a time	Single receptionist
Stage 2: How were the results distributed across the clinicians?	Practice nurse who filed all ‘normal’ results and then to the GP who ordered the test if the result was abnormal	Any GP on duty that day via a daily rota prepared by Office Manager. Nurses also receive results with their name on it	‘Normal’ results filtered to the senior practice nurse by receptionist. ‘Abnormal’ results sent to the GP who had ordered the test	The GP who had ordered the test or their ‘buddy’	The GPs at break time, who then distributed the results themselves according to who ‘knew’ the patient best	The GP who had ordered the test	The GP who had ordered the test and the rest were divided randomly across the GPs	The GP or nurse who had ordered the test via specific chronic disease management clinics (e.g. diabetes, asthma)
Stage 3: How were the results reviewed?	Abnormal results reviewed by GPs in their consulting rooms and normal results reviewed by practice nurses in their consulting rooms	All results reviewed by GPs in their consulting rooms	Results reviewed individually by nurses in their consulting rooms and communally by GPs in a shared computer room during morning coffee break	All results reviewed by GPs in their consulting rooms	All results reviewed by GPs in their consulting rooms	All results reviewed by GPs in their consulting rooms	All results reviewed by GPs in their consulting rooms	All results reviewed by GPs and nurses in their consulting rooms
Stage 4: How did receptionists contact the patients to complete the action?	Patient telephoned by computer operator if a prescription was required and by main reception if an appointment was required	Patient telephoned by member of reception team	Patient telephoned by member of reception team and then send letter if no response	Patient sent letter by member of reception team	Patient telephoned by reception team member and message left if no answer	Patient telephoned by reception team member	Patient telephoned by reception team member	Patients telephoned by a reception team member

#### Stage 1: initial test results management

Across all eight practices, paper results would arrive via NHS internal mail in the morning and were scanned into electronic format, with additional results received electronically. All documents were initially managed by administrative staff who linked them to the patient’s electronic record and assigned them to an appropriate clinician for review. Each of the practices in the study organised this set of processes differently.

In five practices, the scanning of paper results was considered an unspecialised task that could be carried out by any member of the reception team on a daily or weekly rota. The Office Manager of practice 2 explained that a key benefit of this system was that it ‘*makes sure that we’re all up to speed with all the jobs, and so if someone’s off it’s no problem*’. In the other three practices, scanning was the responsibility of a senior member of the reception team who had been trained in the task. In these practices, it was considered more efficient to have just one senior receptionist doing the scanning as it meant that she ‘*knows the job and the patients well*’ (practice 1, receptionist 2).

The larger practices had to process more results each day (typically ~80–100 per day from both scanned and electronic sources). Receptionists in these practices would therefore additionally date-stamp and number all paper results and added them to a log book prior to scanning to ensure that each was carefully accounted for. The smaller practices typically had fewer results to process (~30/day) and so considered the tracking of results unnecessary due to the lower numbers involved:

*The senior receptionist begins opening up the mixed pile of 24 letters and results that arrived this morning. As she opens and checks each result and letter, she explains that she “knows” most of the patients whose results have come through as she deals with most of the chronic disease patients on a regular basis when they make appointments at the front desk […]. Once each letter and result has been opened and added to the pile, she then feeds each sheet of paper through the scanner and electronically adds them to the patient’s notes. (Fieldnotes, Practice 1, 11:14 am, Tuesday 19* ^*th*^ *July 2011)*

#### Stage 2: results distribution across clinicians

Once the results were linked to the patient’s clinical records, they were sent electronically (or ‘workflowed’) to the most appropriate member of the clinical team. This varied across the eight practices, with some initially allocating results to a GP and others to a practice nurse.

In practice 1, all test results were initially workflowed to one of the two practice nurses by the senior receptionist. The nurses screened them to file all of the ‘normal’ results and workflowed the ‘abnormal’ results to the GPs. This system was justified on the basis of historical practice (‘*This system dates back to the 1980s when the nurses used to manage a handful of paper results at coffee time*’ (GP1)) and appropriate use of GP time. All of the GPs in this practice were almost full-time, so it was usually possible for the nurse to send abnormal results to the GP who had requested the test the same day. However, one of the new part-time salaried GPs found this system problematic as she felt that ‘normal’ results did not necessarily mean that no action was required for that patient and that often a normal result can trigger further actions (e.g. further tests). She therefore requested that all of her results were workflowed to her. However, the other partners criticised this as being ‘overly attentive’. In Practices 3 and 8, the receptionists filtered all of the normal results directly to the nurses if their name was on the result. In practice 8, this system was justified on the basis of the high levels of expertise of the nurses who were both prescribers. Also, it was argued that the filtering of normal results from routine chronic disease management testing ensured that the GPs had time to focus on the most complex cases.

In the five other practices, the results were sent to the GP that the receptionist considered most appropriate to deal with it. Decisions about appropriateness varied by practice, based upon their history and internal norms. The majority of the practices prioritised continuity of care, sending the result to the GP who had requested it. However, if the GP who had ordered the test was absent, then ‘most appropriate’ depended on the extent to which the practice also prioritised timeliness and/or equalising GP workload. Practice 4 operated a ‘buddying’ system where each GP had an assigned partner who dealt with their administrative work in their absence. This ensured timely results handling but maintained a level of continuity as each GP also grew to know the patients of their buddy. In contrast, in practice 2 where all of the partners were part-time, the GP who had ordered the test was often not present on the day that the results were returned and there was no formal buddying system. Results were therefore equally divided between the GPs who were present since unnecessary delay was perceived as a potential safety issue.

Across many of the practices, informal systems were also in place if it was unclear who had ordered the test or the name on the test was not a practice clinician (e.g. a locum or retired partners). In the majority of practices, the receptionist would make a decision based on a range of factors, including the perceived speed with which GPs handled results, balanced by judgements about a fair distribution of workload:

*The receptionist on mail and results duty that morning is … distributing them across the four GPs and two practice nurses who are in the practice that morning and afternoon. She explains that one of the GPs in particular was “famous for letting his Docman build up”. She goes on “See, he’s sitting on 208 documents at the moment. He usually tries to get through them at home, but even then they still seem to build up. I suppose we should probably give him less as he’s so slow and we do need to get through these, but we also need to be fair”. She sees from his ‘out of office’ notice that he is absent today and tomorrow, and so decides not to allocate him any today. (Fieldnotes, Practice 2: 11.17 am, Thursday 18* ^*th*^ *August 2011)*

If a GP subsequently considered a result to be better dealt with by a colleague, they would often workflow results to the GP that they considered most appropriate via the reception, even if that meant compromising on handling speed:

*[What] I would tend to do each day is work through the list of results that come directly into my workflow. Some of those will come to me directly but some of them will be triaged to me by the office. Similarly, if a result comes to me that isn’t mine and I want to forward to someone else I send that back to the office and they forward it up to the appropriate person (Practice 4, GP1)*

The distribution of results was therefore determined both by organisational processes and priorities and by individual decisions on the part of receptionists and GPs. Participants expressed a trade-off between better management of results by a clinician who knew the patient or who had ordered the test and the speed with which results were managed including an equitable distribution of work between clinicians. The approach in any one practice or situation reflected perceptions of what mattered most given local context, for example, whether GPs were mostly full or part-time, none of which was explicitly expressed in the formal description of the results handling system (Fig. 1) or in the formal protocols in the three practices that had them.

#### Stage 3: clinician reviews and actions result

Clinicians were workflowed results to review almost every day they worked. In practice 3, the GPs all met at 11 am for coffee in a large room with four computer stations where they worked through their results simultaneously. This allowed them to discuss individual patients and send results to other GPs whom they felt were ‘more appropriate’. In the other seven practices, clinicians usually processed results on their own in their room at variable points in the working day:

*On a busy day I might get 90 or 100 Docman results or items of correspondence to deal with. And you spend a lot of time processing these and quite often by the time you get to look at them it’s half past six or seven o’clock at night when you’re tired and your brain is not working so well. It’s probably not the best time to deal with at least 90 bits of information because each result isn’t just one bit of information, there’s lots of stuff to think about. (GP2 Practice 4)*

Individual GPs frequently commented on the complexity of even apparently normal results. For example, while the system in practice 2 ensured rapid results handling, the GPs raised a concern about the level of clinical attention paid to ‘normal’ results that were not necessarily reviewed by the GP that ordered them:

*Strictly speaking, you should probably find out on every occasion who the result is destined for and redirect it to them so they can mark it as normal. Because, you know, a normal result is you know, isn’t just a normal result, it can mean any number of different things. (Practice 2 GP2)*

In the practices where abnormal results were sent to the GP or nurse who had ordered the test (practices 1, 3, 4, 5, 6, 7 and 8), clinicians frequently reinforced the importance of having sound knowledge of the patient when interpreting test results since an ‘abnormal’ test could be ‘satisfactory’ in an individual patient:

*GP2 was going through the 29 results and mail in her Docman inbox in her consulting room with a cup of coffee following her morning surgery. She explained that many of her patients’ results were ‘abnormal, but perfectly OK for them’. For example, with Patient 11 she checked the blood test result of a female patient of 62 years with urea of 11.6 and creatinine of 141 ‘these results are satisfactory for them, but not normal - they’re borderline. So what I’ll do is keep an eye on her next set of blood results next month’. She then checked ‘no action required’ from the drop-down list and wrote in freetext ‘no change’. (Fieldnotes, Practice 3, 11.17 am, 24.04.12)*

Most tests required no particular action, but the most common forms of action were repeating the test, organising a review appointment and prescribing a medicine. As well as deciding on an action, GPs also had to decide who should take responsibility for that action—themselves, another clinician or receptionists. Who GPs chose to designate to complete actions varied across practices and individual GPs, with the key factors including the perceived urgency of the action, the perceived complexity of any message in the context of the patient’s likely understanding and the time available. Most GPs delegated what they perceived to be simpler actions to reception staff, explaining that it was work best suited to administrators (e.g. ‘*I know some practices the doctors do the actions themselves […] but I don’t see that as a good use of time to me. (Practice 4 GP1))’* and included basic one-line instructions for receptionists to follow such as:

*GP2 selects ‘Appointment with GP’ from the drop-down list and messages reception staff ‘Please arrange a phone consultation to discuss medication change’. (Fieldnotes, Practice 7, 1.40 pm, 13.07.13)*

However, many also stated that there were circumstances when they would take responsibility for action rather than delegating it to a receptionist:

*Patient 29 has a urinary tract infection and requires amoxicillin ‘Usually I would leave it for the girls to prepare the script for me to then sign, but I know that this patient is a worrier, so I would prefer to phone her and prepare the script myself. This way I know that it’s done correctly’. (Fieldnotes, Practice 5, 11.34 am, 08.12.12)*

Both observation and interviews highlighted the complex nature of test result interpretation by GPs and the need to balance ensuring correct interpretation with high and increasing workload. Such decisions were linked both to practice demographics and individual clinicians’ preferences and varied depending on the clinician’s knowledge of a particular patient's circumstances.

#### Stage 4: receptionist actions reviewed result

GPs electronically workflowed results back to the reception team if there was an action they wished them to complete. Although not written down in any of them, all of the practices only contacted patients if a result required further action. If the action involved an appointment or telephone consultation with a doctor, receptionists would usually attempt to arrange the appointment with the GP who had ordered the test rather than the registered or usual GP. Practices varied in terms of how they contacted their patients based on what they believed would ensure effective communication with their patients. Practices 1, 2, 5, 6, 7 and 8 telephoned patients, claiming that it was safest as it ensured that they always reached the correct patient. Practice 2 was located in a highly deprived area, and receptionists regularly stated that many of their patients had literacy issues or did not read their mail and that most would not act on a letter from the practice. Practice 3 had a mixed approach, where they would initially try to telephone, but if that failed, then, they would print a ready-made letters advising them to make an appointment with a GP or nurse. Practice 4 receptionists preferred to send their patient letters as the majority of their patients were elderly, and they perceived that a telephone call would make many patients ‘*panic and want to speak to a doctor that day*’ (receptionist 4) and there were very few on-the-day appointments available. While receptionists generally abided by the practice system, they also regularly made contextual decisions on the best form of communication based on their interpretation of the patient’s circumstances:

*Receptionist 3 telephones [the patient – male, early 30s] on his mobile. [The patient] answers and explains that he is at work and could she call in back in 5 minutes’ time? She phones him back and they agree on an appointment for the following Thursday at 3.20 pm again with GP2. The patient also requests that she send him a letter to remind him of the appointment date and time. Receptionist 3 tells me afterwards: ‘This is not something I normally do. I don’t grudge doing it if they’re old, but it sounds like he’s out and about, so maybe he’ll forget’. (Fieldnotes, Practice 3: 12:37 pm, 03.02.12)*

Individual practices therefore had unwritten but well-understood processes for contacting patients which were generally used and justified in different ways, but GPs and receptionists regularly deviated from these depending on perceptions of the urgency for completing handling of a particular result and knowledge about the needs of particular patients.

## Discussion

This study highlights the importance of informal, contextually appropriate organisational routines for handling test results, which varied between practices depending on how they prioritised different aspects of safety, notably ensuring the handling of results by the most appropriate clinician (typically the person who had ordered the test or knew the patient best) vs. rapid results handling. Ethnographic fieldwork across eight general practices revealed the complex, improvised and context-specific nature of everyday results handling work. While the formal practice-level narratives of results handling routines demonstrated shared understandings of how results handling *should* be done at each stage in the routine (with these occasionally written down in protocols as ‘work as imagined’ [42]), the everyday structure of *how* the routine should be enacted in practice was informally understood (‘work as done’ [42]). This finding also demonstrated that the boundary between what constitutes formal and informal routines is more blurred than that described in the literature, where strict adherence to a single protocol has been shown to create unintended consequences and unsafety [43–46]. In contrast, shared formal practice-level knowledge of general practice results handling routines was mainly learned in situ and held informally at individual and practice levels.

While formal results handling routines were described similarly across the eight study practices, results handling took a range of local forms, with individual practices varying how they balanced the most appropriate person managing the result with the speed with which results were managed depending on historical organisation and internal norms, and patterns of staffing. This type of compromise mirrors the efficiency-thoroughness trade-off (ETTO) faced by workers in complex organisational settings described by Hollnagel, where demands for thoroughness reduce productivity and demands for efficiency reduce quality and precision [47]. Practices in this study managed this trade-off in a variety of ways, with each approach adopted creating its own potential problems or risks (Table 3).

**Table 3:** Test results handling trade-offs

Competency	Examples	Potential risk
Timely management of results	Receptionists allocating results to only the GPs who are present in the practice on that day (practice 2)	Does not allow for additional complexities relating to the patient that the GP ordering the test or the patient’s regular GP might use to make a decision on the result
	Practice nurse screening of ‘normal’ results prior to distribution of ‘abnormal’ results to GPs to optimise use of GPs’ time (practices 1, 3, 8)	Incorporates an additional stage in the results handling process (practice 1). Does not allow for the possibility that a ‘normal’ result may have implications that could be overlooked by the nurse
	Generalist receptionists doing results handling work (practices 2, 4, 5, 6, 7)	May compromise the quality of results handling due to lack of experience of individual receptionists involved in the routine
	Single receptionist amalgamating all results into one generic pile for scanning and workflowing (practices 1, 3)	Potential for error due to lack of systemisation despite small numbers of results being processed
High quality management of results	Single receptionist initially processing test results (practices 1, 3, 8)	Focuses all knowledge of this role into one individual; quality of results processing is potentially compromised if that receptionist is on holiday or off sick
	Single receptionist or team of receptionists manually logging all results received prior to scanning (practices 2, 4, 5, 6, 7, 8)	Laborious and time-consuming
	GP processing the results of tests that they had ordered (practices 1, 3, 5, 6, 7, 8)	Delay in the review and actioning of results if the GP is not available or develops a backlog
	GP processing either the results of the tests that they had ordered or that of their designated ‘buddy’ (practice 4)	Potential for quality of processing to be compromised by relative lack of knowledge of ‘buddy’, plus periods when each buddy has double their usual volume of results to process
	Receptionist telephoning patient regarding abnormal result (practices 1, 2, 5, 6, 7, 8)	Time-consuming as usually requires multiple attempts
	Mixed approach to contacting patient regarding abnormal result combining telephoning and then writing to the patient (practice 3)	Potentially time-consuming but incorporates varied approaches to contacting patient that are potentially more effective than a single approach
	Receptionist writing to patient regarding abnormal result (practice 4)	Uncertainty regarding whether letter has arrived and if patient has read and will act on it, particularly in more deprived areas
	GP screening of both ‘normal’ and ‘abnormal’ results as normal results are not always appropriate to file without further action (practices 2, 4, 5, 6, 7, 8)	Increased volume of results to process; potentially more time-consuming
Being fair whilst being efficient	Receptionist ensuring that all GPs received an equitable allocation of results to process (practices 2, 3)	Different GPs process their results in different ways and at different speeds, which can frequently lead to variations in the speed of results processing (if all are distributed evenly) or quality (if individual GPs are allocated a higher number of results than they are adequately able to process)

Previous studies of safety in complex systems have shown that variability is a feature of all organisational routines that can never be completely eradicated, even when evidence-based care is applied [15]. Our research confirms this finding, highlighting the fact that it is difficult to define one system as necessarily better than any other, not least because practices variably prioritised two legitimate safety outcomes (having the result handled by the most appropriate person and speed of handling). However, this prioritisation was mostly implicit and based upon individual tacit knowledge and local norms [29, 48, 49]. Irrespective of the broad routines adopted by practices, all staff involved in results handling also regularly varied what they did for individual patients depending on the specific context, consistent with Suchman’s [50] proposal that actions are primarily situated (context matters), but that situations are essentially ad hoc. Increased focus should therefore be placed on how best to understand in situ tacit knowledge and appropriate ways of approaching safety improvement for policymakers and practitioners.

This paper also resonates with wider literature demonstrating the importance of intermediaries such as administrative staff in handling and processing decisions in the context of organisational routines [51–53]. Across all of the study practices, receptionists played a pivotal role in results handling decision-making by drawing on context-specific, tacit knowledge that they had developed over time, with this also impacting on patient outcomes. Results handling safety was therefore heavily dependent on the in situ knowledge and close collaboration between GPs and receptionists. Such relationships and practices are not unique to healthcare but have been shown to exist elsewhere, for example in legal [50] and commercial settings [54]. Across these contexts, organisational routines comprise complex interpretative and relational processes and practices, with different socio-technical opportunities and challenges for staff across all levels of the organisational hierarchy.

This ethnographic study also builds on and supports the existing implementation science literature by outlining both the risks and benefits inherent in the top-down implementation of new interventions or guidelines into existing organisational contexts as a basis for improvement in healthcare [55–57]. In particular, previous studies have shown that the broader and more comprehensive approaches to improvement within complex organisational settings are, the more difficult ‘successful’ implementation can be [58, 59]. This study contributes to this literature by ethnographically demonstrating how and why variations in approaches to results handling safety occur in practice. It has also demonstrated the importance of remaining attentive to the characteristics of any safety improvement intervention and its relationship with existing micro-level organisational processes and practices, both prior to and during the implementation process.

### Implications for policy and practice

The paper has focussed on the informal and positive dimensions of safety and has highlighted the need for general practices to develop more proactive approaches to their own results handling safety practices. To date, policymakers and practitioners have focussed on more negative dimensions of results handling safety including the reduction of errors and significant events via ‘best practice’ guidelines and protocols [7]. Current results handling safety improvement initiatives pay insufficient attention to the implementation and delivery of results handling as it is practiced day to day. While protocols and policies rely on static, fixed and generalised circumstances, this paper has shown that safety is relational, dynamic and contested, and that it varies between individuals and across organisational settings. Given the range of approaches to results handling shown in this study, future improvement initiatives within the context of high-volume organisational routines should therefore focus less on the ‘rolling out’ or diffusion of pre-determined technical fixes and more on engaging with the complexity and uncertainty of everyday clinical practice and how local contexts can both contribute to and inform the formal and informal implementation of socio-technical change and innovation [55, 60, 61]. When an intervention is regarded as an integral element of the organisation, there is therefore greater possibility of it achieving longer-term success as it becomes part of the organisational setting and changes with it.

Given the complex and uncertain nature of results handling routines, general practices are also therefore likely to benefit from reflectively engaging in their own in situ improvement. Examples of fruitful approaches that are currently being employed focus on positive deviants [15, 23, 25, 28] or improvement methodologies such as video-reflexive ethnography to highlight existing strengths of practice [62–64]. While everyday systems and processes tend to be implicit and habitual, healthcare improvers have become increasingly interested in surfacing this underlying expertise as a key safety improvement resource in complex systems [26]. Safety improvement interventions should therefore focus on how to maximise practice-level knowledge and understanding of the range of legitimate approaches that are available and the safeties and risks inherent in each. This will enable teams to reflect critically on the safeties and vulnerabilities of their own systems and processes and support practices to recognise their own competencies and vulnerabilities in situ within the context of wider complex system conditions and interactions (e.g. stress/fatigue, usability of technology, design of procedures, social and cultural environments) [44, 65]. This in turn has the potential to open up of new kinds of proactive, contextually appropriate interventions for maximising safety within results handling routines and beyond.

It should, however, also be recognised that the tailoring of care processes to the needs of the individual practice or team member carries its own risks, and teams need to be aware of the trade-offs involved in different formal systems and in the adoption of informal approaches. Practices must therefore achieve a difficult balance between supporting skilled staff in engaging in legitimate and valuable improvisations, whilst at the same time being alert to the risks it may bring. ‘Fail safe’ options, which, for example, provide an audit trail to enable staff to check that actions have been completed may be important, and practices could use training and development opportunities to (re-) evaluate what safety means locally, exploring the strengths of and trade-offs embedded in their own approaches and actively considering alternative, contextually meaningful approaches to safe patient care. The key implication for intervention developers [35] is that complex interventions to improve the safety of results handling which focus on the high-fidelity delivery of relatively standardised results handling systems are unlikely to be optimal in all practices, and it is likely to be more appropriate to deliver an intervention to practices that allows them to tailor a system appropriate to their local context [66] and which motivates and enables them to reflexively monitor their work to ensure that tailoring remains appropriate [67].

## Strengths and limitations

This study employed ethnographic methods to examine results handling in eight UK general practices. The key strength of this work was the large number of hours of detailed observation alongside in-depth interviews in multiple carefully sampled field sites by a non-clinical researcher who was trained in anthropology. This in-depth comparative approached allowed for a detailed examination of the informal, tacit everyday practices of primary care teams that are usually taken for granted by practice members themselves, and which an interview study may overlook. Despite being conducted in a relatively small number of practices, this study will have applicability beyond the eight general practices in which it was conducted. While there are likely to be many more ways of achieving results handling safety beyond those identified in this study, we anticipate that the informal competencies and trade-offs identified will resonate with healthcare practitioners and safety improvers across a range of national and international contexts.

## Conclusions

This paper further adds to the literature examining the value of the positive characteristics of safety embodied in healthcare organisational routines, where both ‘success’ and ‘failure’ are products of the same informal results handling processes [27, 68]. To date, much patient safety research and improvement work has focussed on measuring and managing more negative dimensions of safety such as risk and error. As practices are encouraged to work together in more formalised ways [69], practices sharing premises and seeing one another’s patients will need to develop new routines that are transferrable and meaningful in a variety of settings. This study contributes to a newer body of research evidence that examines the in situ knowledge required to maintain safety and mitigate risk within and across complex organisational settings [13, 27, 70, 71]. This has the potential to inform new approaches to intervention development and implementation focussing on enhanced deliberation of the safety of existing routines and practices and has highlighted a need for further research to examine how best to improve results handling alongside other complex inter-professional organisational routines.

## Abbreviations

GP: General practitioner
IT: Information technology
NHS: National Health Service
